# Personalised nutrition technologies: a new paradigm for dietetic practice and training in a digital transformation era

**DOI:** 10.1111/jhn.12746

**Published:** 2020-03-16

**Authors:** M. Abrahams, N. V. Matusheski

**Affiliations:** ^1^ Faculty of Management, Law & Social Sciences University of Bradford Bradford UK; ^2^ Qina Ltd Olhao Portugal; ^3^ Nutrition Science and Advocacy DSM Nutritional Products Parsippany NJ USA

**Keywords:** behaviour change, digital health, digital literacy, mHealth, personalised nutrition, technology

## Personalised nutrition in patient‐centred care

It has recently been estimated that one in five early deaths worldwide is associated with poor dietary habits ^(1)^. Addressing this societal challenge through dietetics practice will require substantial human resource investment. However, according to the World Health Organization, there is currently a substantial shortage of healthcare workers, which is expected to increase over the coming decades ^(2)^. Because of this, it is important to develop an understanding of the potential ways that new technologies and digital tools can help to increase the impact of dietetics. This will create value not only for the individual patient, but also as a scalable approach to helping individuals develop improved dietary habits and transition to a service that is based on prevention and self‐care.

Patient‐centred care is a cornerstone of modern dietetics practice ^(3)^. A key tenant underlying this approach is the individualisation of guidance based on the patient’s specific needs and, in a broad sense, treating the patient and not the disease ^(4)^. In parallel, personalisation has recently developed as a trend in the consumer nutrition and wellness area ^(5)^. Numerous apps, programs, platforms and plans are now aimed at delivering a personalised experience for the user based on profiling of an individual’s demographics, genotype, nutritional intake and status, anthropometrics, lifestyle behaviours, and/or preferences ^6^, ^7^. Several definitions have been put forth for personalised nutrition, including those that deal primarily with genetic differences, and others that include much broader concepts, including phenotypic, psychosocial and behavioural aspects of individualisation ^8^, ^9^, ^10^, ^11^. In this editorial, we employ a recently proposed definition of personalised nutrition to describe approaches that ‘use individual‐specific information, founded on evidence‐based science, to promote dietary behaviour change that may result in measurable health benefit’ ^(12)^. By leveraging a holistic definition, we can consider how different aspects of personalisation can be of greatest benefit and can be most effectively leveraged for the individual patient, which can be appreciated by all dietitians.

The scientific evidence for personalised nutrition is growing. Substantiation continues to emerge that personalised nutrition can provide added value beyond conventional approaches. In the clinical setting, there has been increased recognition of the importance of implementing nutritional screening and intervention ^(13)^. For example, individualised nutrition assessments and provision of tailored nutritional support in patients at nutritional risk have been shown to significantly improve clinical outcomes, including patient survival ^(14)^. In a broader wellness context, several important gene–diet interactions were found to influence the response to dietary weight‐loss interventions in the landmark DIOGENES trial ^(15)^.

More holistic approaches, leveraging personalised information based on both genotypic and phenotypic variation, have also been promising ^(16)^. A recent study in older Dutch adults found that the provision of personalised advice, based on dietary intake, genetic and physiological information, resulted in increased resiliency and motivation, and decreased body fat percentage and hip circumference ^(17)^. In the future, we expect to see even more research investment in Personalised ‘algorithm‐based’ approaches. For example, clinical trials are currently underway aiming to validate a microbiome‐based personalisation approach for blood sugar management ^18^, ^19^. Likewise, an ongoing collaboration between Stanford University and Massachusetts General Hospital recently published a pilot study (PREDICT) and is now conducting a large observational study (PREDICT2) to measure individual metabolic responses to foods, with the aim of developing a commercial platform ^(20)^. In the Nutrigenomics, Overweight/Obesity and Weight Management Trial (NOW Trial), the effects of a lifestyle intervention employing personalised genetic testing and behavioural advice will be compared with the same intervention with population‐based advice ^(21)^. However, for many commercial platforms, their benefit has yet to be established in randomised controlled trials. Challenges still exist in terms of replicability of results, diversity of population groups included ^(22)^, as well as scientific validation and accuracy of products currently available ^(23)^. It has become clear however, that behaviour change is the common denominator underpinning successful personalised nutrition approaches for which dietitians are well versed, trained and experienced.

## A practical personalised nutrition framework

Considering the high level of consumer interest in personalised nutrition, it is not surprising that many commercial personalised nutrition programs have arisen. However, each approach varies regarding the information that it collects about an individual, and which recommendations arise. Because of this, it is important to develop a framework for assessing whether a given personalised nutrition platform can offer real benefits, or whether some alternative should be recommended. An interdisciplinary expert group ^(12)^ recently developed a set of 10 ‘guiding principles’ for personalised nutrition that can support such an assessment (Box 1). These principles can be of equal value for those developing and for those using or implementing a technology‐enabled personalised nutrition program. Using such a framework can help determine whether a personalised nutrition approach is credible and would be expected to deliver results for an individual.

Box 1 **Guiding principles for personalised nutrition.**

1. Define potential users and beneficiaries
2. Use validated diagnostic methods and measures
3. Maintain data quality and relevance
4. Derive data‐driven recommendations from validated models and algorithms
5. Design personalised nutrition studies around validated individual health or function needs and outcomes
6. Provide rigorous scientific evidence for an effect on health or function
7. Deliver user‐friendly tools
8. For healthy individuals, align with population‐based recommendations
9. Communicate transparently about potential effects
10. Protect individual data privacy and act responsibly

Despite a rapid rise in availability, the integration of digital tools into daily dietetic practice remains low amongst dietitians. In a survey of dietitians in Canada, Australia and the UK, 63% of respondents reported using a mobile health app in their practice, primarily for informational and patient self‐monitoring purposes ^(24)^, yet very few are used for behaviour change ^25^, ^26^. Another study in Australia demonstrated poor eHealth readiness in terms of advocacy, although there was an improvement with respect to attitudinal, aptitude and access to eHealth readiness ^(27)^. A recent multi‐national survey showed that dietitians who had adopted personalised nutrition innovations demonstrated higher levels of self‐efficacy, lower perceptions of risk and higher usefulness, and also assigned a higher importance of technology to dietetic practice, compared to those who had not ^(28)^. Interestingly, dietitians who had integrated personalised nutrition technologies perceived themselves to be entrepreneurs, bringing another dimension to how we may need to address digital transformation and organisational change in a modern data‐driven healthcare service ^(29)^. As a result of these advances in both science and technology, it is important for the practising dietitian to increase their awareness, knowledge, digital literacy (in terms of artificial intelligence and genomics) ^(30)^, professional skills and comfort level with respect to the digital solutions that power these personalised recommendations through big data analytics, machine learning and artificial intelligence (AI).

## The growing role and need for the next generation of dietitians

Although the guiding principles are an excellent reference point for those aiming to develop personalised nutrition solutions, the growing role for the next generation of dietitians is clear. Digital solutions will not replace dietitians because of the crucial value that we bring in terms of personal relationship building and behaviour change ^(31)^. However, dietitians who do not adopt or sufficiently understand new technologies may run the risk of being replaced. As a profession, we need to address this new reality at all levels of personalisation. Dietitians can play an important role in new initiatives and product development to ensure that digital products are scientifically valid, inclusive, equitable, accessible, explainable and representative.

The opportunities for dietitians as we move into the fourth industrial revolution are limitless and include those outlined in Box 2.

Box 2 **Opportunities for dietitians in the fourth industrial revolution.**

1. **Acquiring new skills** in bias validation, algorithm development, data management and analytics, as well as workflow, and unlocking new business models.
2. **Identifying new career opportunities** that can uniquely combine the best of humanity, society, sustainability and technology to impact health outcomes for all
3. **Creating a new value proposition for dietitians** as we leverage our nutrition domain expertise with digital literacy and a focus on prevention
4. **Learning a new language** in terms of digital technologies and regulation that transcends borders as technology increases our reach

## Strategic considerations for digital transformation in dietetic practice

In a modern healthcare system, which is transitioning to one that is participatory and personalised, we need to ensure that we are equipped with the right skills, knowledge and mindsets for this shift. These skills include inclusive leadership, developing an entrepreneurial mindset ^28^, ^32^, data management and digital literacy ^(30)^. At present, the area of tech‐enabled personalised nutrition receives little attention in the dietetic curriculum ^(33)^. To our knowledge, with the exception of genomics, new technologies such as AI, machine learning and neural networks are not currently covered in the dietetic curriculum. This is concerning, considering that the recent survey cited above demonstrated that most **Registered Dietitians (RDs)** did not consider technology to play an important role in dietetic practice ^(28)^. However, we know that students are interested ^(33)^, which highlights that there is indeed a gap between consumer demand and current dietetic awareness.

## Conclusions

The time is right for dietitians to take the lead in the digital transformation of healthcare services, with nutrition and lifestyle playing a vital role in the prevention of noncommunicable diseases. New personalised nutrition technologies that are based on science, and are inclusive and accessible, provide new ways of delivering care and reaching key groups to support them in lasting behaviour change. Dietitians have a unique opportunity to be a guiding voice, a reality check and a key resource for the creation and delivery of new solutions and healthcare models. To become the reference professionals for a data‐driven future that is already here, we need to address where we are as a profession in terms of our inclusive leadership, and ensure that our digital and entrepreneurial literacy skills are truly at the forefront of change.
